# Fat necrosis in the Breast: A systematic review of clinical

**DOI:** 10.1186/s12944-019-1078-4

**Published:** 2019-06-11

**Authors:** Narges Vasei, Azita Shishegar, Forouzan Ghalkhani, Mohammad Darvishi

**Affiliations:** 1 Department of Surgery, Besat Hospital, AJA University of Medical Sciences, Tehran, Iran; 2 Infectious Diseases and Tropical Medicine Research Center (IDTMRC), Department of Aerospace and Subaquatic Medicine, AJA University of Medical Sciences, Tehran, Iran

**Keywords:** Breast, Fat necrosis, Oncoplastic surgery, Mammography, MRI

## Abstract

Breast fat necrosis (FN) originates from aseptic fat saponification, which is a typical lipid cyst or a spiculated lesion called mammographic presentation which mimics malignancy. In order to avoid biopsy, it would be necessary to identify the spectrum of fat necrosis appearances. A systematic research was conducted in October 2018 by using PubMed, MEDLINE, Embase, Google Scholar databases and Google to search for science literature published after 2004. Therefore, the aim of this systematic review, it is that the FN can provide radiologists, surgeons, and oncologists with better insight and help them manage the condition efficiently.

## Introduction

### Features of Fat necrosis

Known as a benign, non-supportive inflammatory process, breast fat necrosis (FN) occurs due to iatrogenic breast trauma. Fat necrosis is an inflammatory, sterile process which has roots in fat aseptic saponification. In this regard, blood and tissue lipase contribute to this disorder. Some of the common causes of FN are radiotherapy, surgery or trauma, particularly one associated with anticoagulation therapy. It is mostly associated with post-operative or radiation therapy [1]. It is difficult to distinguish the clinical and radiographic appearance of breast fat necrosis from that of malignancy. For this purpose, biopsy is recommended by the research community. Some of the main clinical features of fat necrosis are irregular masses with skin retraction and multiple smooth round nodules [2]. The palpable abnormality is often superficial and periareolar. In some rare cases, they can result in tenderness, bruising, skin tethering, dimpling and nipple retraction. In addition, breast abnormalities may remain unchanged, enlarge, regress, or resolve [1]. Taboada et al., [3] showed that in about 47.4% of cases fat necrosis would be appeared as cystic masses which shown in Table 1 (In accordance with Prasanti et al. [4]).

**Table 1:** Fat necrosis various Imaging appearances (In accordance with Prasanti et al.[4])

Cystic lesions are associated with mural nodules	Anechoic cystic mass with enhanced through transmission	Anechoic masses which envelop in shadow	Composite masses in shape of cyst
15%	16.6%	15.8%	47.4%

### Fat necrosis pathology

**Fig. 1:**
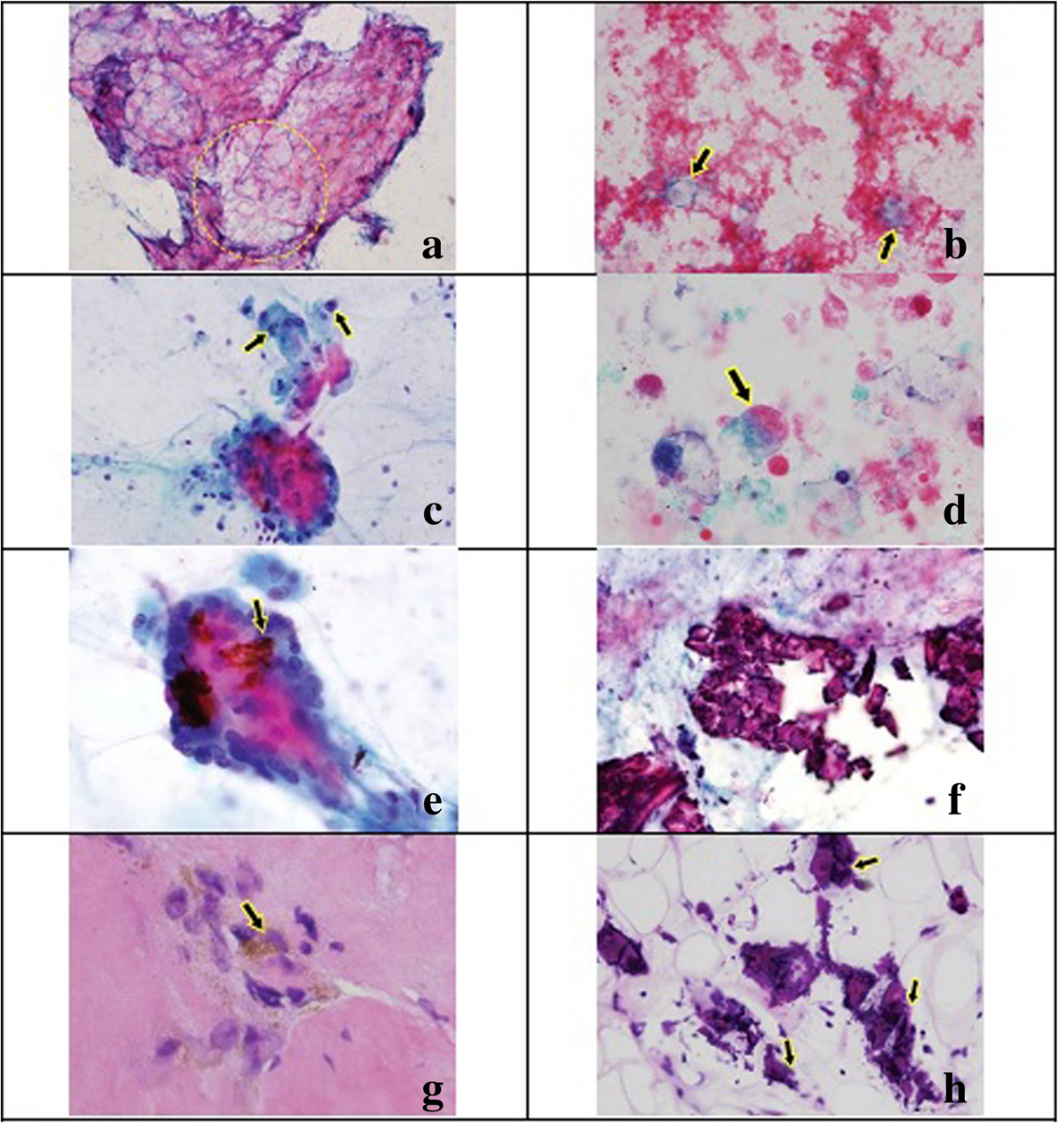
Different levels of fat necrosis (Derived in accordance with Jorge et al. [6]). **a** Primary level of fat necrosis indicates fragments of adipose tissue. **b** Primary level of fat necrosis indicates individual adipocytes. **c** Medium level of fat necrosis indicates infiltration by histiocytes. **d** Medium level of fat necrosis indicates conglomeration of RBCs referred to as “myospherulosis”. **e** Late stage of fat necrosis indicates single multinucleated giant cell. **f** Late stage of fat necrosis indicates calcifications (are common in late stage of fat necrosis). **g** Late stage of fat necrosis indicates macrophages containing hemosiderin. **h** Late stage of fat necrosis indicates calcifications

Fat necrosis may result in firmness and induration on gross pathology. Thin capsules or lipid cysts may emerge in fibrosis. Older lesions may emerge as oil cysts. It should be noted that these ring-like calcifications may also emerge in the wall [5]. Fat necrosis may occasionally be seen as dense masses characterized by skin thickening. It is said that outpouring of blood into the parenchyma may result in swollen trabecular framework in the breast. This is usually associated with disruption of fat cells. This destruction may form intracellular vacuoles which are usually filled with necrotic material (Fig. 1). Fibroblasts, multinucleated giant cells and lipid- laden histiocytes (‘fat-filled macrophages’ or ‘foam cells’) accumulate between cyst-like areas (Fig. 1). [3]

### Breast oil cysts

**Fig. 2:**
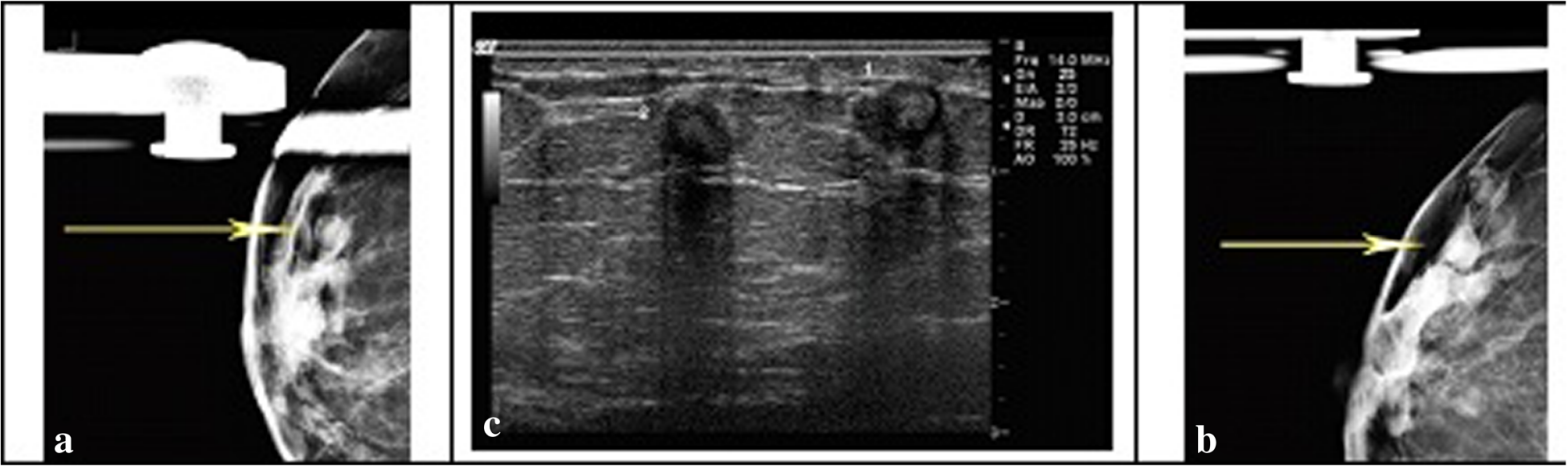
Craniocaudal mammograms and right breast mediolateral oblique. **a** and **b**) show round masses with radiolucent centers at the site of palpable finding. **c**) Ultrasound of the right breast at site of palpable finding demonstrate two hypoechoic round masses with central echogenicity with associated posterior acoustic shadowing (Derived in accordance with William et al. [19])

Oil cysts which appeared in imaging of the breast may point out the lesions within the breast where a focal fat necrosis scope would be separated via fibrous tissue. Oil cysts of breast are mostly consisting of pure neutral fat. One of the main forms of necrosis is breast fat necrosis which could be specified by the action on fat via digestive enzymes [6, 7]. In fat necrosis, the glycoprotein enzyme lipase frees fatty acids from triglycerides. The released fatty acids then will be combined with calcium and would form soaps which become visible as white pale deposits [8]. During the process of palpation, oil cysts are soft and moving tissues. These cysts are often representing approximately all of fat necrosis and need only a modest aspiration by application of syringe and a hypodermic 18-gauge needle 18 Gauge. The appearance of sticky oil might be white or yellow and commonly cannot be syringed via slim needles. A lipid cyst is an oval mass with a thin bright smooth border. The cyst fibrous rim might be calcifying or not [9, 10]. Anyway, when the fibrous rim of the cyst is calcified, it might be seen in face or profile (Fig. 2). Infrequent known forms of lipid cysts such as cysts which have serous-hemorrhagic contents or fat-fluid levels and/or aspergillums and also cysts which show advanced opacification (Fig. 1) [11].

### Fat necrosis etiology

Peri-menopausal women suffer mostly from breast fat necrosis. This disorder is capable of mimicking breast cancer radiologically and clinically. It is usually secondary to trauma but is sometimes idiopathic. Trauma can have accidental or iatrogenic nature. Seatbelt trauma is one example of accidental injury. Some of the main iatrogenic events are breast surgery (lumpectomy, reduction, or augmentation) [12]. Lumps were seen in many patients suffering from trauma. Seatbelts may probably be one of the causes of such lumps. The aetiological factors include lumpectomy, radiotherapy, breast reconstruction, cyst aspiration, reduction mammoplasty, implant removal, biopsy, trauma, anticoagulation. Some of the other causes include Weber-Christian disease, granulomatous angiopanniculitis and polyarteritis nodosa. [13]. The cause remains unknown in some patients.

### Fat necrosis pathogenesis

Necrosis and apoptosis are two types of cell death which have different biochemical and morphological features. Necrosis is considered a kind of cell death, while apoptosis is defined as the process of complicated cell death. These two are triggered by numerous stimuli including pathogens, ischemia, irradiation, cytokines and heat. Moreover, mitochondria, kinase cascades and death receptors have a role to play in these processes [14]. Cytosolic components may trigger an inflammatory reaction in necrosis. However, cell membranes isolate these products in apoptosis. Both processes are observed in fat necrosis of the breast [15]. Necrotic components may lead to greater inflammation and worsen the condition. Fibrinogen is secreted in the interstitial area by damaged vessels. Thrombin converts fibrinogen to active fibrin. The oil cyst is characterized by the round lesion and wall calcification. The oil cysts can be reliably diagnosed by ultrasound [16, 17].

### Fat necrosis detecting

It is necessary to investigate the reasons why fat necrosis emerges on imaging techniques because it may prevent misinterpretations regarding the imaging findings. Moreover, it is necessary to understand the variable appearances of fat necrosis in order to help radiologists enhance their accuracy, particularly for analyzing and interpreting fat necrosis. Fat necrosis is capable of creating numerous findings. MRI is in in good agreement with fat necrosis histology [18]. Despite the fact that mammography is a specific technique, ultrasound is an efficient tool for the diagnosis of fat necrosis. MRI may indicate unusual peripheral enhancement in fat necrosis. However, fat necrosis appearance may not be distinguished from adverse lesions. Despite the sheer fact that PET-CT is not the sole tool for the diagnosing fat necrosis and breast cancer, it is widely used in for diagnostic purposes [14].

### Mammography test

Some of the common fat necrosis findings include coarse calcifications, cysts, micro-calcifications, asymmetries and so on. Within initial stages fibrosis is not much extensive and is attendant with capsules which appear in oval or round shapes [16]. It is while lesions which are older would be seen as calcifications in the wall (Fig. 2). Older lesions may emerge as oil cysts that have ring-shaped calcifications in their wall [5]. Fat necrosis may occasionally be seen as unusual masses characterized by skin thickening. It is said that outpouring of blood into the parenchyma may result in swollen trabecular framework in the breast. This is usually associated with disruption of fat cells [14].

### Sonography test

Fat necrosis may be associated with solid or cystic masses. Solid masses are characterized by well-circumscribed margins, and may distort the breast parenchyma [2]. Fat necrosis appearance ranges from complex intra-cystic masses to solid nodules. The oil cyst is associated with round lesion and wall calcification. Sonography can be used to diagnose oil cysts. In some rare cases, hyperechoic masses may represent malignancy. The sonographic appearance of an oil cyst could easily determine that which kind of breast cysts is that. It’s while in some cases when the appearance of oil cyst is worrying a needle aspiration should be done for testing the content of the cyst [2].

### MRI test

**Fig. 3:**
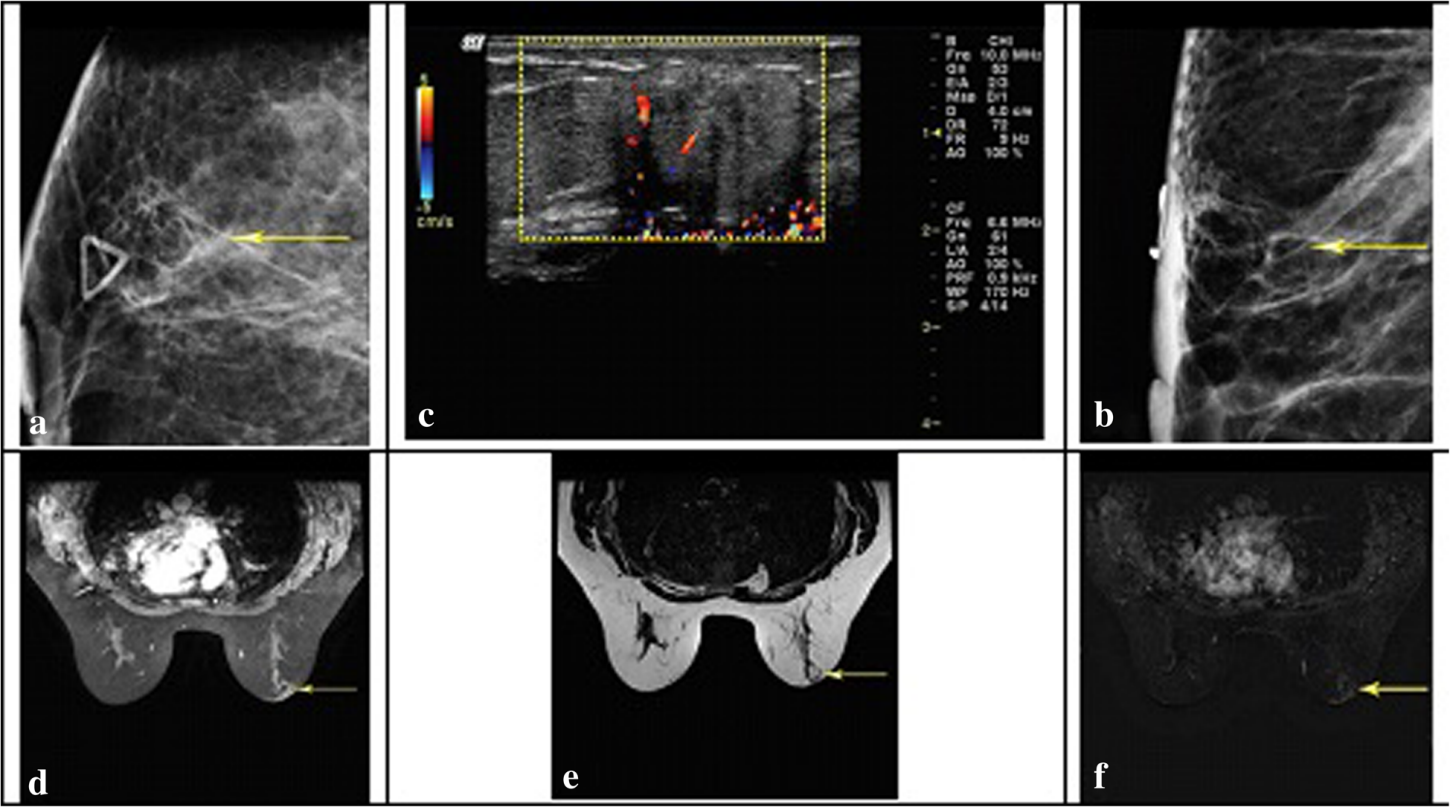
Craniocaudal projections and right breast mediolateral oblique. **a** and **b**) show a radiolucent lobular mass at site of palpable mass (arrow). **c**) Targeted ultrasound at site of palpable mass demonstrates a lobular heterogeneous hypoechoic mass with posterior acoustic shadowing. **d**) Axial T1-weighted fat saturation after gadolinium. **e**) T2-weighted nonfat saturation, and **f**) subtraction images that indicate a mass at 11 o’clock in the right breast anteriorly that follows fat signal on all sequences with thin rim enhancement (Derived in accordance with William et al. [19])

Fat necrosis is capable of producing various findings. MRI images are in good agreement with fat necrosis histology. Siderophages may trigger a diffuse decrease in signal intensity on images. Fat necrosis is constituted of lipophagic granulomas which are found on T1-weighted images [17]. It is quite difficult to distinguish lipophagic granulomas from malignancy on MRI. Biopsy is needed to confirm the diagnosis of such lesions. MRI may be capable of showing unusual enhancements in fat necrosis. However, fat necrosis appearances may be may not be distinguished from malignant lesions [19]. As mentioned earlier, fat necrosis is often emerges as fat elsewhere in the breast which is necessary for the diagnosis of FN [20]. (Fig. 3).

### CT scan test

CT Scan is capable of showing areas of fat necrosis. It can allow the researchers avoid any misunderstandings of the findings. CT is based upon fibrosis, liquefied fat and inflammation. These components are mainly seen in fat necrosis. Liquefied fat manifests itself on CT Scan as low coefficients of attenuation, while fibrosis is represented as soft tissue coefficients. Fat necrosis mimics the appearance of breast cancer on MRI, appearing as spiculated enhancement or a cystic lesion [14]. F^18^-FDG PET/CT can somehow help identify local recurrence, although it is not a recommended method [21]. It is essential to consider the histological results along with the imaging and clinical findings. Moreover, a multidisciplinary team needs to be involved in this process. The common imaging features of fat necrosis are summarized in Table 2 [1, 2, 14, 15, 17, 18, 20].

**Table 2:** Common imaging features of fat necrosis

MRI	(1) Depending on amount of inflammatory reaction, liquefied fat, and degree of fibrosis, fat necrosis produces a wide spectrum of findings on MRI. Magnetic resonance images correlate well with the histology of fat necrosis.
	(2) Depending on the intensity of the inflammatory process, it may show enhancement after the administration of IV paramagnetic contrast material.
	(3) Lipid cyst, round or oval mass with hypointense T1-weighted signal on fat saturation images are the most common types of appearance.
	(4) It is usually isointense to fat elsewhere in the breast. (v) Compared with surrounding fat, “black hole” sign is marked hypointensity on STIR images.
	(5) MRI is associated with thin, thick, irregular or spiculated enhancement.
Mammography	The mammographic appearance of fat necrosis includes normal appearance, discrete round or oval radiolucent oil cyst with thin capsule, thickening and deformity of skin and subcutaneous tissue, focal mass, and ill-defined spiculated mass. Oil cysts may be associated with uniform continuous eggshell calcification. There may also be multiple clustered pleomorphic micro-calcifications suspicious of malignancy. The most common mammographic findings are dystrophic calcifications, followed by radiolucent oil cysts.
Ultrasound	Common features of fat necrosis on sonography are increased echogenicity of subcutaneous tissue, as an anechoic cyst with posterior acoustic enhancement, hypoechoic mass with posterior acoustic shadowing, solid mass, cyst with internal echoes, normal appearance or cystic mural nodule and architectural distortion
CT	(1) Liquefied fat can manifest low attenuation coefficients.
	(2) Similar to fibroglandular tissue or linear densities resembling fibrous bands, fibrosis has attenuation.
	(3) Inflammation enhances followed by contrast injection.
PET-CT	(1) Secondary to presence of metabolically active inflammatory cells, fat necrosis may increase FDG uptake.
	(2) It is associated with intense activity in the setting of TRAM flap reconstruction.

### Mammoplasty surgery

It is essential to evaluate the severity and fat necrosis prevalence with each procedure. Moreover, in order to address the defects of breast-conserving therapy, good grafts need to be selected. The oncoplastic surgery provides a better cosmetic outcome and controls local tumor. It is also safe to investigate early-stage breast cancer [22]. After conservative sessions of therapy, breast cancer may occur years after the treatment. Patients may undergo ultrasound (US) and mammographic examinations during the follow-up period [23]. Treated breast is prone to several modifications after surgery and radiation therapy. This can complicate the interpretation of images, particularly when local recurrence is suspected. Despite the fact that MRI is not included in routine follow-up, it is still used in clinical practices. Compared to the conventional imaging examinations which discriminates between postsurgical tissue modifications and tumor recurrence, is it very sensitive [24].

As a combination of BCS, since the early-1970s, BCT has been regarded as a standard therapy for breast cancer in early stage. According to several clinical trials, obvious evidence shows that breast-conserving therapy is associated with long-term survival rate [25, 26]. In spite of the fact that BCT started years later in Japan, some Japanese pioneers used that technique in the mid- 1980s [27]. BCT witnessed a rapid growth. As reported by the registry of the Japan Breast Cancer Society (2015), BCT is now used to treat 50% of breast cancer patients.

The long-term success of BCT is influenced by the rate of local control and the preserved breast’s cosmetic appearance. There are several strategies involved in this field. Tumor burden diagnosis [28] and breast margin pathological diagnosis [12, 29] are among the main strategies. Moreover, primary chemotherapy can be used for conservation of breast, particularly when breast cancer is in advanced stage [30]. There are several types of BCSs that can be used to obtain good cosmetic results, including lateral tissue flap, inframammary adipofascial flap [31], the moving window technique [32]. In this study, various procedures have been applied for replacing defects of partial resections. These categories are mainly known as oncoplastic breast-conserving surgeries. Fat necrosis can negatively affect the quality of life of the patients because of poor cosmetic result and pain. Unfortunately, fat necrosis cannot be efficiently evaluated because there are no standard systems for grading [33]. Breast surgeons need to explain the severity and of frequency fat necrosis to the patients. For this purpose, it is highly recommended that annual mammography and physical examinations be performed, particularly for those who have undergone BCT [34].

It is still challenging to manage fat in practice. It can still be difficult to diagnose female breast fat necrosis, even via advanced diagnostic instruments. Cancer recurrence and fat necrosis need to be differentiated, especially in patients undergone surgery of breast conservation. In certain cases, diagnosis confirmation can be done through the needle core biopsy. There has been an increase in the number of early-stage breast cancer due to the mammographic screening programs [35]. BCS is a standard care in breast cancer patients at early stages [24]. According to research, compared to mastectomy, BCS offers equal rates of survival (over 20 years) [36]. Reducing postoperative deformities and promoting safe breast conservation are possible through BOS [37, 38]. After surgery and radiation therapy, the breast tissue undergoes various changes. This can complicate the interpretation of image during the period of follow-up, particularly when there is suspect of local recurrence [39]. According to the evidence, MRI has not yet been recommend in the following up the patients who have received BCS. According to the current guidelines, MRI is not recommended for follow-up of breast cancer in asymptomatic patients. During the period of follow-up, many mammographic and ultrasound (US) examinations are performed for patients [13, 39]. When there are margins of post-resection positive tumor, Evaluation of the suspicious recurrences emerging on either mammography/US or clinical examinations can be helped by MRI. Moreover, MRI can also be used to screen patients who have a high recurrence risk following treatment of breast cancer [40]. In responding inflammatory postoperative reactions, the strong enhancement of resection margins can affect early postoperative MRI. Once the breast-conserving therapy is completed, MRI cannot exclude possible residual tumor [41]. Compared to conventional imaging investigations, MRI is far better in discriminating tumor relapse from postsurgical tissue modifications [42]. Premenopausal women need to be examined on the 6th to 13th day of the menstruation. This can help minimize the risk of false positives [43]. Diffusion-weighted imaging (DWI) has recently been paid great attention in the clinical settings. DWI-MRI is based upon measuring the random Brownian motion of water molecules within a tissue. Diffusion can be particularly used in tumor characterization [44].

### Fat necrosis occurrence after BCS

**Fig. 4:**
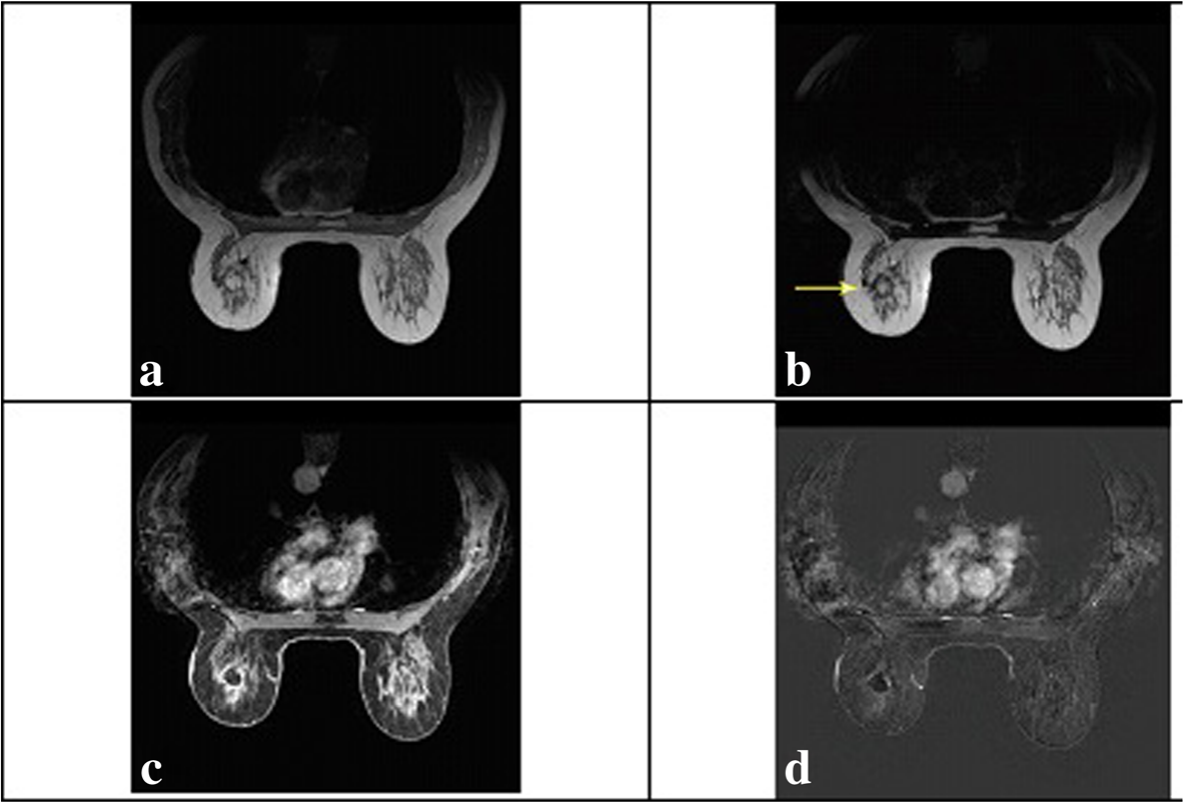
A patient who shows a mass in the left breast which follows fat signal on all sequences (arrow). **a**) Axial T1-weighted nonfat saturation, **b**) T2-weighted nonfat saturation, **c**) T1-weighted fat saturation after gadolinium and **d**) subtraction images (Derived in accordance with William et al. [19])

As a benign inflammatory process of adipose tissue, fat necrosis affects menopausal women to a great extent. Surgery, radiotherapy, or traumas are some of the main causes of FN. The patients can be affected by iatrogenic, penetrating or blunt trauma. Women with breast hypertrophy also suffer from physical symptoms including psychosocial problems and back pain. These problems can lead to activation of lipolytic enzymes and rupture of blood vessel [1]. Adipose tissue consists of triglyceride-containing cells. Fatty acids that release from triglycerides and enter the interstitial space lead to formation of a complex with calcium. This can cause aseptic fat saponification [2]. Results of imaging and biopsy following BOS and breast lumpectomy were compared by Dolan et al. [45]. According to the results, fat necrosis rate following BCS on US and clinical examinations was 15 and 18%, respectively. According to the results of their study, patients who had undergone BOS need more US examinations and following biopsies compared to patients who had undergone lumpectomy. In the majority of cases, this can mainly be attributed to FN developing after BOS. FN is often known as an asymptomatic condition, but patients may suffer from skin thickening, erythema, ecchymosis, and a palpable mass. A radiolucent rounded image was indicated by the clear mammographic image. Accordingly, calcifications may result in the suspicion of disease relapse. It should be pointed out that US image can sometimes be misleading and indicate an apparent hypoechoic area with posterior acoustic shadowing and blurred margins [46–49] (Fig. 4). Depending on the stage of the process, FN may have different presentations.

Patients expect a natural shape after BOS [50]. Known as a reconstructive and aesthetic technique, Lipo injection is widely used in breast surgery [51]. According to the Fat Graft Task Force of the American Society of Plastic Surgeons (ASPS) [7], the effectiveness of the procedure was evaluated in patients. Only 37 complications (12.7%) were reported, the most common of which was lipo necrosis in 16 cases (5.7%) [7]. The difficulties in assessing patients undergoing breast Lipo injection following BOS were fundamentally associated with FN [49]. Patients may suffer from palpable and painful mammary swelling. Given fat- post-contrast graphic and suppressed sequences, it is possible to distinguish between breast cancer relapse and lip necrosis [52].

### Thickening of skin and breast subcutaneous edema

Given the small vessel damage, skin thickening is often expected to occur, particularly after BOS. Edema can affect the whole breast after radiation therapy. All signs of inflammation can be revealed through clinical examination. Some of the main sign include red skin or tissue stiffness, and soreness [40, 41]. The US image can help diffuse structural dishomogeneity and provide evidence for skin thickening. If the thickness of the skin is more than 4 mm, it is necessary to use MRI with fat suppression for evaluation purposes. During the first 6 months after radiation therapy, the above mentioned alterations become more obvious. In majority of the patients, the alteration may decrease or stabilize within a 2–3- year period [40, 41]. Further research is required to investigate the increase in skin thickening because the patient may likely be prone to inflammatory breast cancer [53].

### Reactivation of tumor

During the first 5 years after treatment, breast cancer may be reactivated, particularly following conservative surgery and radiotherapy [38, 54]. Local tumor reactivation can be considered a relapse of tumor cells in the site of tumor, in spite of surgery. However, regional tumor reactivation refers to the extra spread of the initial cancer out of the breast which involves the axillary lymph nodes [55]. Patients who have undergone BOS are more likely to be affected by local tumor relapse, compared to patients after mastectomy [54]. Given the fact that the conventional imaging techniques are inconclusive or in conflict with some clinical indicators, MRI can help detect the suspected reactivation more efficiently. Additionally, MRI is an appropriate tool for evaluating the extent of residual tumor in women following lumpectomy with positive or close resection margins [56]. It is of great importance to distinguish a tumor reactivation from scar tissue after BOS. Compared to mammography, MRI is a very sensitive tool (75–100%) [57]. Based on MRI, some patients need to undergo a follow-up course. It may not be easy to detect ipsilateral recurrent tumors via first-step breast investigations (i.e., US and mammography). Dynamic contrast-enhanced MRI can be used to accurately detect the extent, location, and presence of tumor reactivation [43].

## Discussion

The breast fat necrosis is a frequent benignant situation that could induce a broad range of mammographic findings such as tissue lumps, calcifications with various morphology and size, oily cysts and localized skin thickening [58]. A collection of fused macrophages known as foreign-body giant cells, lipid-laden macrophages, occupying interstices penetration via plasma cells are systematically present. Fat saponification leads to vacuoles formation which then would be surrounded via macrophages. Therapeutic management via fibrosis would begin at the edge and finally might be replaced with the whole area or evacuate a persevering cystic cavity [16]. In some cases, the cyst content might do not mantain in its uniform radiopacity, as a consequence of simulating an abscess, simple cyst or solid lesion. Distortion of rim of conglomerate masses would cause the cyst wall edges to become irregular. When the content of the cyst is a combination of both blood and the liquid part of blood (serum), the mixture could layer on horizontal-beam sidelong films. In such a cases based on highly expressive clinical findings, periodic follow up of patients are recommended instead of carrying out a biopsy. In such cases when there is a nodule in a lipid cyst and incidental calcifications may be present, biopsy would be recommended [16, 42].

Fat necrosis has several clinical and imaging features which may not be sometimes easy to distinguish from malignancy [13]. This condition may be asymptomatic. Moreover, sometimes the physician can detect the pathologic condition only through mammography. [59, 60]. The mammographic spectrum of appearances of fat necrosis should be recognized in order to avoid biopsy. Given the growing advances in surgical technique and materials, patients believe that the natural contour, shape, and symmetry should be reconstructed at the culmination of the breast reconstructive process [58]. Unfortunately, secondary contour deformities can still occur and negatively affect the expectations of patients. Despite the fact that fat graft is now a popular technique, it is still associated with some major concerns.

## Conclusion

As one of the commonest types of cancer, breast cancer affects women. BOS aims to obtain radical tumor excision. For this purpose, MRI can be used to detect local breast cancer recurrence. The evidence does not yet recommend MRI in the follow-up of patients who have undergone BCS. According to the current guidelines, MRI is not recommended for breast cancer follow-up in asymptomatic patient. In this period, US and mammographic examinations are performed. MRI can be used as an evaluation tool for residual diseases. Despite the fact that fat grafting is an efficient tool, it may develop a significant risk of fat necrosis/oil cyst. Physicians need to adequately inform the patients about the sequelae of fat grafting and the possible need for future ultrasounds and biopsy. Further research is needed to investigate the radiologic features of palpable nodules with ultrasound and development of standards. The characteristic descriptors need to be evaluated in order to efficiently fat necrosis. It is necessary to differentiate fat necrosis from malignancy in patients who have had fat grafting.

## Data Availability

Not applicable.

## Abbreviation

ASPS: American Society of Plastic Surgeons
BCS: breast-conserving surgery
BCT: breast-conserving therapy
BOS: breast oncoplastic surgery
DWI: Diffusion-weighted imaging
EUSOMA: European Society of Breast Cancer Specialists
FN: at necrosis
MRI: magnetic resonance imaging
US: ultrasound
