# Correction to: Fat necrosis in the breast: a systematic review of clinical

**DOI:** 10.1186/s12944-019-1097-1

**Published:** 2019-08-01

**Authors:** Narges Vasei, Azita Shishegar, Forouzan Ghalkhani, Mohammad Darvishi

**Affiliations:** 1 Department of Surgery, Besat Hospital, AJA University of Medical Sciences, Tehran, Iran; 2 Infectious Diseases and Tropical Medicine Research Center (IDTMRC), Department of Aerospace and Subaquatic Medicine, AJA University of Medical Sciences, Tehran, Iran

**Correction to: Lipids Health Dis (2019) 18:139**

**https://doi.org/10.1186/s12944-019-1078-4**

Following publication of the original article [1], the authors reported an incorrect reference found in Figs. 1, 2, 2 and 4 legends.

The corrected figure legends are shown below.

Fig. 1 Different levels of fat necrosis (Adapted with permission from Taboada et al. [3]). **a** Primary level of fat necrosis indicates fragments of adipose tissue. **b** Primary level of fat necrosis indicates individual adipocytes. **c** Medium level of fat necrosis indicates infiltration by histiocytes. **d** Medium level of fat necrosis indicates conglomeration of RBCs referred to as “myospherulosis”. **e** Late stage of fat necrosis indicates single multinucleated giant cell. **f** Late stage of fat necrosis indicates calcifications (are common in late stage of fat necrosis). **g** Late stage of fat necrosis indicates macrophages containing hemosiderin. **h** Late stage of fat necrosis indicates calcifications

Fig. 2 Craniocaudal mammograms and right breast mediolateral oblique. **a** and **b**) show round masses with radiolucent centers at the site of palpable finding. **c**) Ultrasound of the right breast at site of palpable finding demonstrate two hypoechoic round masses with central echogenicity with associated posterior acoustic shadowing (Adapted with permission from Kerridge et al. [19])

Fig. 3 Craniocaudal projections and right breast mediolateral oblique. **a** and **b**) show a radiolucent lobular mass at site of palpable mass (arrow). **c**) Targeted ultrasound at site of palpable mass demonstrates a lobular heterogeneous hypoechoic mass with posterior acoustic shadowing. **d**) Axial T1-weighted fat saturation after gadolinium. **e**) T2-weighted nonfat saturation, and **f**) subtraction images that indicate a mass at 11 o’clock in the right breast anteriorly that follows fat signal on all sequences with thin rim enhancement (Adapted with permission from Kerridge et al. [19])

Fig. 4 A patient who shows a mass in the left breast which follows fat signal on all sequences (arrow). **a**) Axial T1-weighted nonfat saturation, **b**) T2-weighted nonfat saturation, **c**) T1-weighted fat saturation after gadolinium and **d**) subtraction images (Adapted with permission from Kerridge et al. [19])
